# CD26 Induces Colorectal Cancer Angiogenesis and Metastasis through CAV1/MMP1 Signaling

**DOI:** 10.3390/ijms23031181

**Published:** 2022-01-21

**Authors:** Lui Ng, Sunny Kit-Man Wong, Zheng Huang, Colin Siu-Chi Lam, Ariel Ka-Man Chow, Dominic Chi-Chung Foo, Oswens Siu-Hung Lo, Roberta Wen-Chi Pang, Wai-Lun Law

**Affiliations:** Department of Surgery, Li Ka Shing Faculty of Medicine, The University of Hong Kong, Pokfulam, Hong Kong; sunnykitman@gmail.com (S.K.-M.W.); mr.zen.hwang@gmail.com (Z.H.); siuchi88@gmail.com (C.S.-C.L.); ariel115@gmail.com (A.K.-M.C.); ccfoo@hku.hk (D.C.-C.F.); oswenslo@yahoo.com (O.S.-H.L.); lui_ng@hotmail.com (R.W.-C.P.)

**Keywords:** CD26, colorectal cancer (CRC), metastasis, CAV1, MMP1

## Abstract

CD26 has been reported as a marker for colorectal cancer stem cells endowed with tumor-initiating properties and capable of colorectal cancer (CRC) metastasis. In this study, we investigated the functional effect of CD26 on CRC angiogenesis and metastasis, and the potential underlying mechanism. The functional effects of CD26 overexpression or repression were determined by a wound healing experiment, and cell migration and invasion assays in vitro and in mouse models. Differentially expressed genes regulated by CD26 were identified by genome-wide mRNA expression array and validated by quantitative PCR. CD26 functionally regulated CRC cell migration and invasion in vitro and angiogenesis and metastasis in vivo. Genome-wide mRNA expression array and qPCR showed that MMP1 was up-regulated in CD26+ subpopulation, and a subsequent experiment demonstrated the regulatory effect of CD26 on MMP1 in CRC cell lines with CD26 repression or overexpression. Furthermore, overexpression of CAV1 abrogated the CD26-regulated MMP1 induction in CRC cell lines. This study demonstrated the functional roles of CD26 in inducing CRC migration, invasion, angiogenesis and metastasis and identified the potential involvement of MMP1 and CAV1 in such process. CD26 is an attractive therapeutic target for combating tumor progression to improve the prognosis of CRC patients.

## 1. Introduction

Colorectal carcinoma (CRC) is the third most prevalent and the fourth most lethal type of cancer worldwide. In fact, a significant portion of CRC patients develops synchronous or metachronous liver metastases [1]. The five-year survival rate of CRC patients with liver metastases is less than 10% although with the advanced therapeutic regiments nowadays [2]. Understanding the underlying mechanism of metastasis is important for the development of novel therapeutic strategies.

Our previous works have successfully identified a subpopulation of CD26+ CRC cancer stem cells (CSCs) associated with the development of distant metastasis, enhanced invasiveness and chemoresistance in colorectal cancer [3]. CD26+ CRC cells were uniformly present in both the primary and metastatic tumors in colorectal cancer patients with liver metastasis [3]. Furthermore, in patients without distant metastasis at the time of presentation, the presence of CD26+ cells in their primary tumors predicted distant metastasis on follow up. Isolated CD26+ cells, but not CD26− cells, led to the development of distant metastasis when injected into the mouse cecal wall [3]. In addition, CD26+ cells were also associated with enhanced invasiveness and chemoresistance. These findings suggested that these CD26+ cells are the CSCs responsible for the metastatic capacity of CRC cells [3]. Following the identification of CD26+ CSCs in CRC, we and the others subsequently showed that high CD26 expression in CRC specimens is associated with higher TNM staging, the development of metastasis and is a predictor of poor prognosis after resection of CRC [4,5,6]. These results consistently supported the poor prognosis associated with the presence of CD26+ cells in tumors. However, the functional role and underlying mechanism of CD26 in CRC need to be further explored. 

In addition, one of the crucial steps for tumor progression and, consequently, metastasis, is the secretion of factors from tumor cells into the microenvironment which then act on the stromal cells to promote the growth of new blood vessels in the process known as angiogenesis [7]. During angiogenesis, new vessels emerge from existing endothelial-lined vessels to promote the degradation of the vascular basement membrane and remodel the extracellular matrix (ECM), followed by the migration and proliferation of endothelial cells and the generation of new matrix components [8]. Numerous studies have demonstrated the key participation of matrix metalloproteinases (MMPs) in the degradation of ECM components, tumor cell migration into the near tissue, as well as promoting tumor growth and spread through the capillary endothelium and neovascularization [9]. 

In this study, we showed that the knock-down of CD26 impaired CRC cell migration, invasion, angiogenesis and liver metastasis. Molecular study revealed that CD26 induced MMP1 level in CRC, possibly through its negative regulation on CAV1. These findings demonstrated the mechanism of CD26-induced CRC metastasis and might be an attractive target for the treatment of CRC.

## 2. Results

### 2.1. Transient CD26 Repression Impaired Epitheliasl–Messenchymal Transition (EMT) Pathway in CD26+ Colorectal Cancer (CRC) Cells

**Figure 1:**
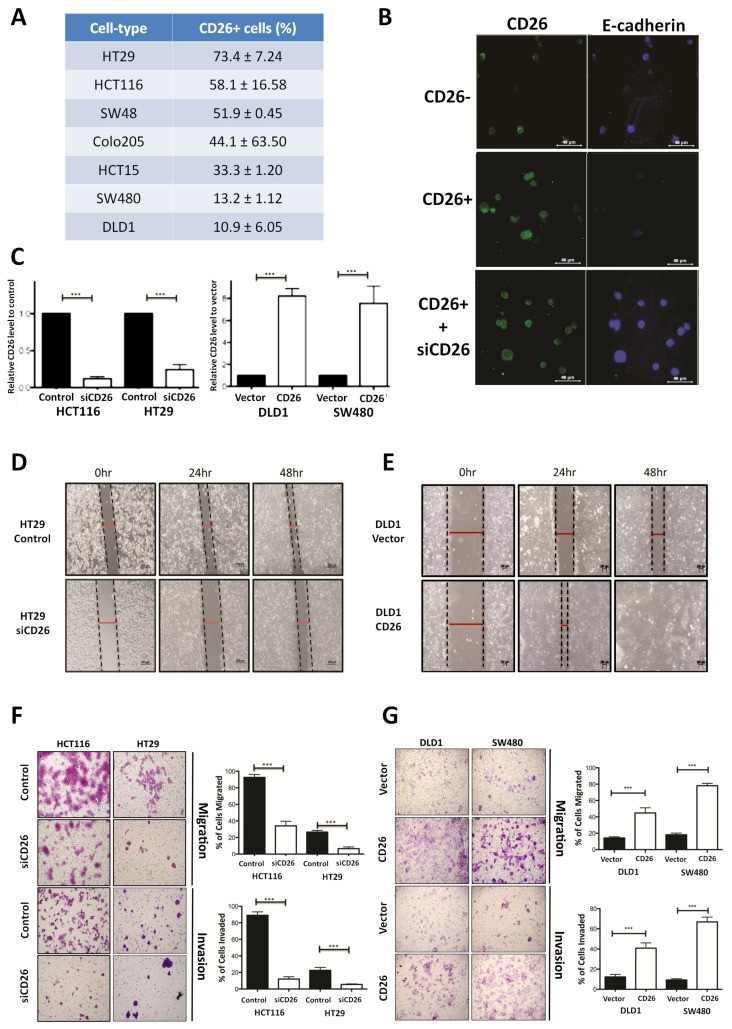
CD26 functionally induces cell migration and invasion in colorectal cancer (CRC) cells. (**A**) Percentage of CD26+ cells in different CRC cell lines. (**B**) Immunofluorensce showing level of CD26 (green) and E-cadherin (blue) in HCT116 fluorescence-activated cell sorting (FACS)-sorted CD26-, CD26+ and CD26+ cells with CD26-siRNA (siCD26) transfected. CD26+ cells showed lower E-cadherin in comparison to CD26− cells, indicating induced EMT in CD26+ cells, whereas such effect was abrogated by transient repression of CD26 by siRNA (siCD26). The scale bars correspond to 40 µm. (**C**) Relative CD26 level in (left) CD26-siRNA transfected HCT116 and HT29 cells when compared to control transfected cells and (right) CD26 overexpressed DLD1 and SW480 cells when compared to vector control cells. (**D**) HT29 cells with transient CD26 knock-down by siRNA (siCD26) showed decreased wound-healing ability compared to siRNA-control transfected cells (control). The scale bars correspond to 200 µm. (**E**) DLD1 cells with transient CD26 overexpression (CD26) showed increased wound-healing ability compared to vector control (vector). (**F**) HCT116 and HT29 cells with transient CD26 knock-down by siRNA (siCD26) showed decreased migration and invasion ability compared to control-treated cells (control). The scale bars correspond to 200 µm. (**G**) DLD1 and SW480 cells with transient CD26 overexpression (CD26) showed increased migration and invasion ability compared to vector control (vector). *** denotes *p* < 0.0001.

We firstly determined the proportion of CD26+ cells in a series of CRC cell lines by flow cytometry (Figure 1A). To investigate whether CD26 functionally induces CRC metastasis, we determined the level of E-cadherin, which is the hallmark of epithelial markers, in fluorescence-activated cell sorting (FACS)-sorted CD26+ and CD26− HCT116 cells. As shown in Figure 1B, the proportion and intensity of CD26 staining in CD26+ cells were higher than CD26− cells. In contrast, the staining of E-cadherin was lower in CD26+ cells when compared to CD26− cells, suggesting that the CD26+ cells were undergoing EMT process. In addition, when CD26 was repressed in CD26+ cells by transient transfection of CD26-siRNA (siCD26), the level of E-cadherin staining was induced. These results suggested that CD26 functionally induces EMT in CRC cells as reflected by reduced E-cadherin level.

### 2.2. CD26-Induced Colorectal Cancer (CRC) Migration and Invasion In Vitro

To further investigate the functional role of CD26 in CRC, its expression in CRC cell lines was induced or repressed, and the resultant effect on cell migration and invasion in vitro and metastasis in vivo was assessed. HT29 and HCT116 which have a relatively higher proportion of CD26+ cells (Figure 1A) were used for CD26 repression experiments by transient transfection of CD26-siRNA (siCD26). On the other hand, DLD1 and SW480 which have lower proportion of CD26+ cells were used for CD26 overexpression experiments by transfection of CD26 expression plasmids (CD26). The efficiencies of CD26 knock-down or overexpression were shown in Figure 1C. Wound healing experiments were initially performed to determine the effect of CD26 repression or overexpression on cell migration of HT29 and DLD1 cells, respectively. Figure 1D shows the wound closure after 24 and 48 h for HT29-siCD26 and HT29-control cells. The wound distance was larger in siCD26 cells at 24 and 48 h compared to control cells. On the other hand, the wound distance was smaller for CD26-overexpressed DLD1 cells at 24 and 48 h compared to vector control cells (Figure 1E).

We further compared the migratory and invasive ability of CD26-manipulated cells using the migration and invasion transwell assays, respectively. As shown in Figure 1F, transient CD26 repression by CD26-siRNA (siCD26) in HCT116 and HT29 cells led to a significant decrease in migration and invasion abilities. When compared to control cells, HCT116-siCD26 cells showed significant repressed migration (from 92.3% to 34.3%, *p* < 0.0001) and invasion potential (from 89.3% to 13.0%, *p* < 0.0001), whereas HT29-siCD26 cells also showed a significantly lower percentage of migrated (from 26.0% to 6.3%, *p* < 0.0001) and invaded cells (from 21.7% to 5.3%, *p* < 0.0001). On the other hand, overexpression of CD26 in DLD1 and SW480 cells significantly induced their migration and invasion abilities (Figure 1G). Comparing to vector control cells, DLD1-CD26 overexpressing cells showed induced migration (from 14.2% to 44.8%, *p* < 0.0001) and invasion abilities (from 12.3% to 40.8%, *p* < 0.0001), whereas SW480 cells also showed significant induction in percentage of migrated (from 18.2% to 78.2%, *p* < 0.0001) and invaded cells (from 9.3% to 66.8%, *p* < 0.0001).

### 2.3. CD26 Knock-Down Impaired Colorectal Cancer (CRC) Metastasis and Angiogenesis in Mouse Model

**Figure 2:**
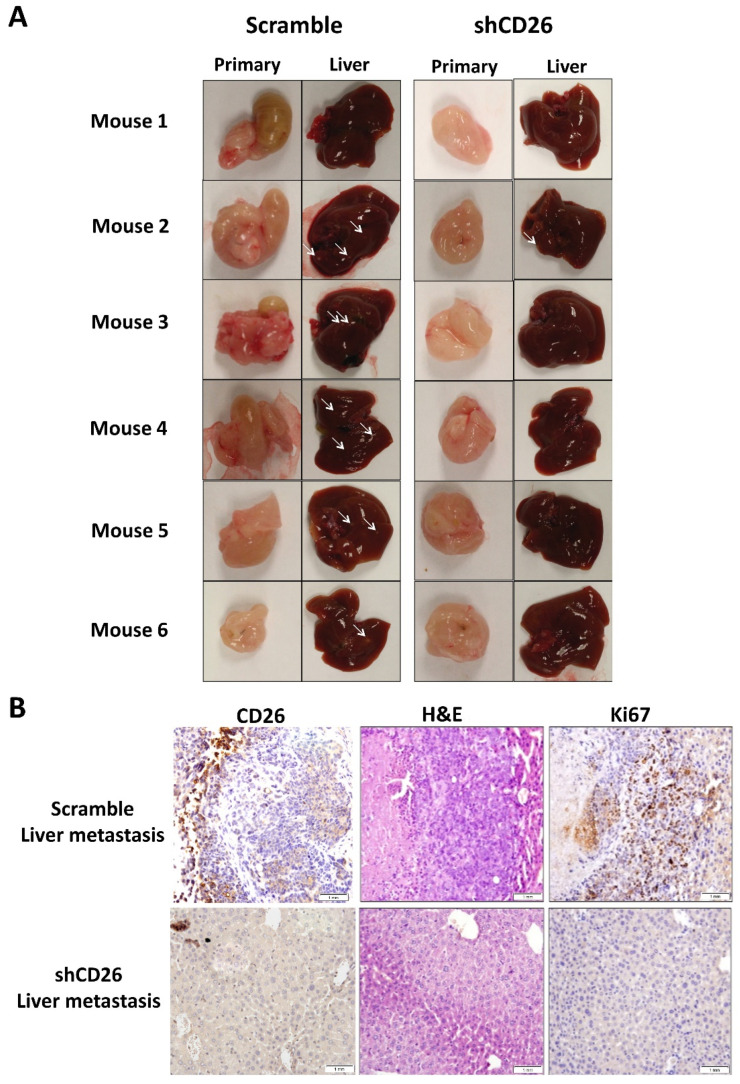
CD26 knock-down impairs CRC metastasis in mouse model. (**A**) HT29 cells with CD26 stably knock-down by retroviral-based method (shCD26) showed reduced ability to form metastatic liver nodules in immunodeficient mice when compared with the scramble group. White arrows indicate a potential tumor nodule on the liver. (**B**) Representative picture showing CD26 (**left**) level in liver metastasis of shCD26 and scramble groups. H&E (**middle**) and anti-human Ki67 (**right**) staining confirmed presence of liver metastasis. The scale bars correspond to 1 mm.

The effect of stable CD26 knock-down on in vivo metastatic potential of CRC cells was also investigated. CD26-repressed HT29 stable cells by CD26-shRNA (shCD26), and their respective control cells (scramble) were orthotopically implanted into the cecal wall of NOD-SCID mice. The development of primary tumor and liver metastasis was investigated after 1 month. All the mice developed primary tumor at cecum (Figure 2A). Visible nodules were observed in the liver of 5 out of 6 mice in the HT29-control group, suggesting the presence of liver metastasis, whereas only 1 out of 6 mice in the HT29-shCD26 group showed such nodule (Figure 2A). Figure 2B shows the level of CD26 in liver metastasis of scramble and shCD26 cells. The development of liver metastasis was confirmed by H&E and anti-human Ki67 staining (Figure 2B). These results revealed that the metastatic potential of CRC cells was significantly impaired by CD26 repression (Fisher’s exact test, *p* = 0.003).

**Figure 3:**
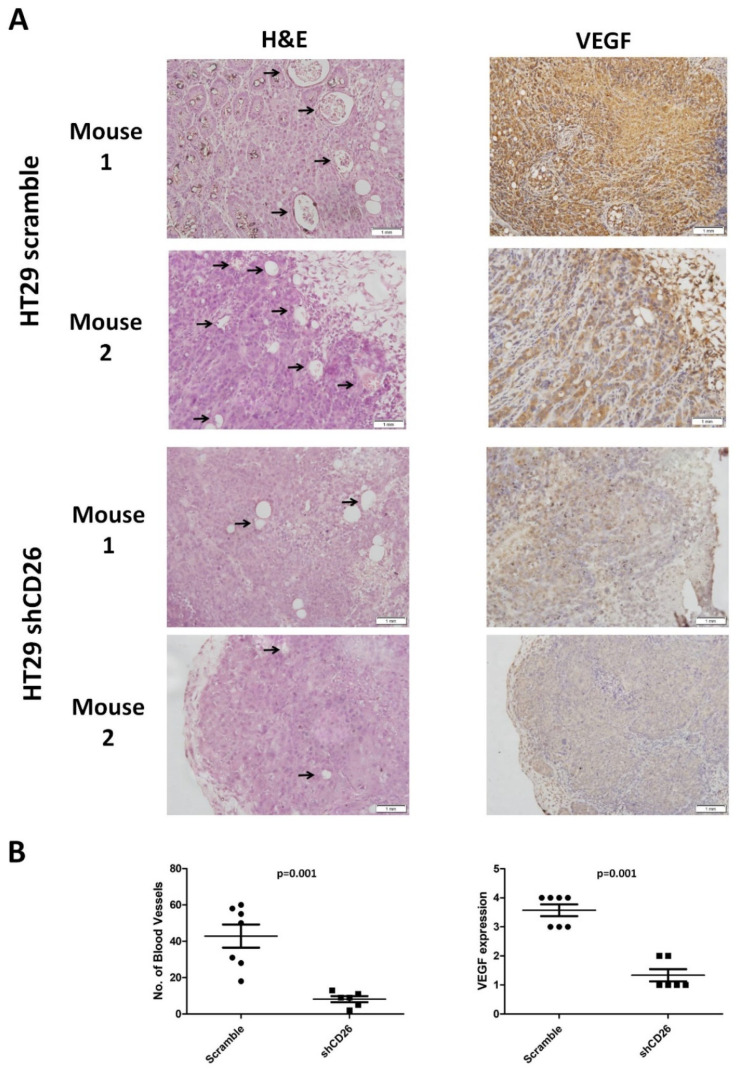
CD26 knock-down impairs CRC angiogenesis in mouse model. (**A**) Two representative photographs from scramble control and shCD26 group showing the blood vessels (indicated by arrows) formed in hematoxylin and eosin (H&E)-stained sections of primary tumor (**left**) and the expression of VEGF which is an important signaling protein involved in both vasculogenesis and angiogenesis (**right**). The scale bars correspond to 1 mm. (**B**). Dot plotted graphs showing lower number of blood vessels (**left**, average of 8 random fields at 40× magnification) and lower VEGF level (**right**) in the shCD26 group.

Furthermore, to evaluate the effect of CD26 on angiogenesis in a CRC tumor, we measured the number of blood vessels in the primary tumor from the shCD26 group and the scramble control group. The total number of blood vessels was determined in H&E-stained sections in 8 random fields in high-power microscopic fields (×40) by counting vascular structures based on typical morphological appearance and the presence of the counted vascular lumen and intraluminal red blood cells (indicated by arrows). The number of blood vessels was significantly reduced in shCD26 group when compared to scramble control group (Figure 3A,B). In addition, the expression of VEGF which is an important signaling protein involved in both vasculogenesis and angiogenesis was also significantly reduced in the shCD26 group (Figure 3A,B). These results demonstrated that CD26 repression impaired angiogenesis in CRC.

### 2.4. Gene Expression Profiling Revealed Elevated MMP1 in CD26+ Cells

In order to identify the underlying molecular mechanisms associated with CD26-mediated tumor metastasis and angiogenesis, we performed genome-wide mRNA expression array to identify dysregulated genes between CD26+ and CD26− HT29 cells. A total of 7902 differentially expressed genes were identified, including 3255 upregulated genes and 4647 downregulated genes. As shown in Table S1, certain genes related to pluripotency including *SOX2, SOX4, CD133, CXCR4* and *CD44* [10]; and genes associated with epithelial to mesenchymal transition (EMT) such as *ZEB2* and *SNAI1* were upregulated. These preliminary findings reaffirmed the association of CD26+ CRC cells with stem cell properties and activation of EMT process.

**Figure 4:**
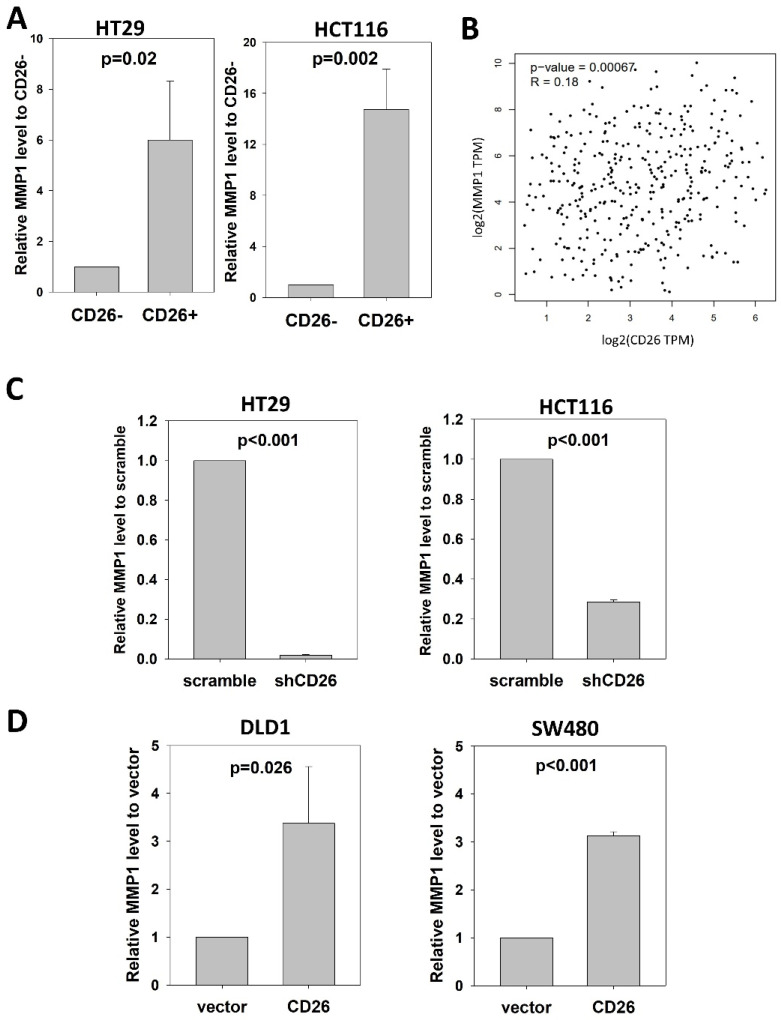
CD26 regulates MMP1 expression in CRC cells. (**A**) Relative MMP1 level in CD26− and CD26+ cells of HT29 and HCT116 cell lines. (**B**) CD26 level was positive correlated with MMP1 in CRC specimens from TCGA database. (**C**) Stable CD26-repressed cells (shCD26) of HT29 and HCT116 showed lower MMP1 level. (**D**) Stable CD26-overexpressed cells of DLD1 and SW480 showed higher MMP1 level.

Notably, we found consistent upregulation of *MMP1* in CD26+ HT29 cells (Table S1). MMP-1 is a collagenase that degrades ECM, specifically targeting type I, II and III collagens, the major components of the interstitial stroma, and is required for angiogenesis [11]. In CRC, MMP-1 expression correlates with the level of invasion, lymph node involvement, metastasis and poor prognosis [12,13]. To confirm the association between CD26 and MMP1, we performed qPCR to determine the level of *MMP1* in CD26+ and CD26− HCT116 and HT29 cells. As shown in Figure 4A, the level of *MMP1* in CD26+ cells was 6- and 14.7-fold higher than in CD26− cells for HT29 and HCT116, respectively.

We further analyzed whether *CD26* was associated with *MMP1* in CRC specimens (Figure 4B). Expression level of *CD26* and *MMP1* in CRC specimens from TCGA was analyzed at GEPIA (http://gepia2.cancer-pku.cn/#index (accessed on 23 November 2021)), which showed that *CD26* was positively correlated with *MMP1* level in colon and rectum cancer (R = 0.18, *p* = 0.00067).

The positive association between *CD26* and *MMP1* suggests that MMP1 is involved in CD26-induced tumor progression effects in CRC cells.

### 2.5. CD26 Functionally Regulated MMP1 Expression through CAV1

To investigate whether CD26 functionally induced *MMP1* level in CRC cells, we perform qPCR to determine the level of *MMP1* in shCD26 and scramble control of HT29 and HCT116 cells. MMP1 was significantly reduced following CD26 repression, which was ~0.02- and ~0.28-fold when compared to scramble control for HT29 and HCT116 cells, respectively (Figure 4C). In addition, we studied the effect of CD26 on MMP1 by determining the *MMP1* level in DLD1 and SW480 CD26-overexpressed and vector control cells. Following CD26 overexpression, the level of *MMP1* was ~3.4- and ~3.1-fold induced when compared to vector control for DLD1 and SW480 cells, respectively (Figure 4D).

**Figure 5:**
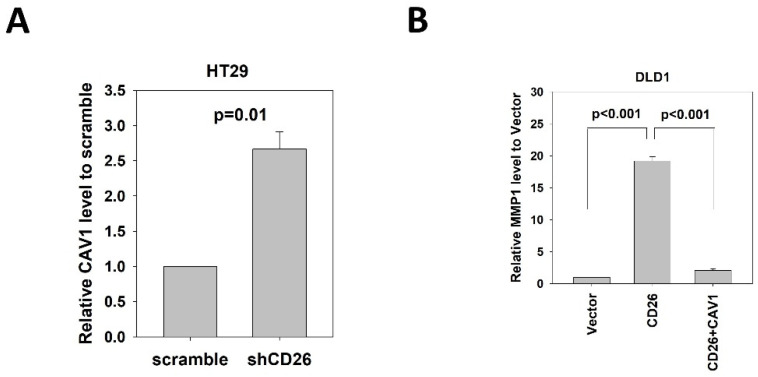
CD26 regulates *MMP1* expression through repression of *CAV1* in CRC cells. (**A**) QPCR revealed that *CAV1* level was induced in HT29 cells following stable repression of CD26 (shCD26) compared to control (scramble transfected cells). (**B**) Relative MMP1 gene level in control (vector), CD26 overexpressing cells (CD26) and CD26 overexpressing cells with transient overexpression of CAV-1 of DLD1 cells. The induction of *MMP1* by CD26 overexpression was abrogated by following CAV1 overexpression.

CAV1 has been reported to negatively regulate the expression of MMP1 [14]. Our mRNA expression array demonstrated lower *CAV1* level in FACS-sorted CD26+ HT29 and HCT116 cells when compared to the CD26− counterparts (Table S1). In addition, our qPCR results showed that *CAV1* was up-regulated in the HT29-shCD26 cells when compared to the scramble control cells (Figure 5A), indicating that CD26 functionally repressed the level of *CAV1*. Hence, we hypothesized CD26 regulated MMP1 level via CAV1. In line with our hypothesis, transient overexpression of CAV1 in DLD1 stable CD26 cells abrogated the *MMP1* induction effect by CD26 overexpression (Figure 5B).

These findings demonstrated that CD26 induced the expression of MMP1 through repressing CAV1.

## 3. Discussion

Colorectal cancer (CRC) is one of the most common malignancies and leading causes of cancer death in the world [1]. Approximately 50% of patients with CRC develop metastasis and hence the prognosis is poor [2]. Therefore, understanding the biological mechanism of CRC metastasis is important for developing new therapeutic approach to treat CRC patients. Recent evidence demonstrated in several types of cancer that cancer stem cells (CSCs) are a subpopulation of cells within the tumor bulk needed for cancer initiation and progression and possess the chemoresistant property [15,16,17,18,19]. Our team previously demonstrated that a subpopulation of CD26+ cells was associated with the development of distant metastasis in colorectal cancer through binding to extracellular matrix components such as fibronectin and collagen, and regulating the expression of EMT markers [3]. Following the identification of CD26+ CSCs in CRC, we and the others subsequently showed that high CD26 expression in CRC specimens is associated with higher TNM staging, development of metastasis and is a predictor of poor prognosis after resection of CRC [4,5,6]. These results consistently supported the poor prognosis associated with presence of CD26+ cells in tumors. Yet, the functional significance of CD26 in CRC metastasis is unexplored. Hence, this study aims to investigate the functional role of CD26 and the underlying mechanism in CRC metastasis.

Our results showed that in addition to the biomarker role of CD26+ in CSC stratification and prognosis for post-operative CRC patients, CD26 indeed functionally induces the metastatic potential of CRC cells. Our in vitro experiments demonstrated that transient overexpression of CD26 in CRC cell lines induced their migration and invasion abilities, whereas transient repression of CD26 resulted in the decreased mobility, migratory and invasion potentials of CRC cells. Moreover, the in vivo ability of HT29 cells to regulate angiogeneis and liver metastasis was drastically impaired when CD26 was stably repressed. These findings suggested that CD26 is an attractive therapeutic target for combating tumor progression. Actually, CD26 inhibitor, also known as DPP4 inhibitor or gliptin, are a class of oral hypoglycemic drugs that antagonize the enzyme DPP4 and are approved to treat diabetes mellitus type 2 (DM-II). We and others recently reported that CRC patients with concurrent DM-II demonstrated better prognosis when treated with CD26 inhibitors [20,21,22]. Further research on CD26 and its inhibitors is warranted to provide further support for targeting CD26 in CRC treatment.

This study showed for the first time the association of CD26+ cells with tumor angiogenesis and its regulatory effect on *MMP1* expression. MMP-1 is widely reported to dissolve ECM, and subsequently initiate and promote angiogenesis [23]. One of the reported mechanism is MMP1 induces the expression of the vascular endothelial growth factor receptor 2 (VEGFR2) and endothelial cell proliferation, stimulates serine/threonine protein kinase MARK2 and activates the transcription factor NF-κB and hence vascular remodeling and angiogenesis [24]. In another study, inhibition of MMP1 by chemical inhibitor or neutralizing antibodies impaired angiogenesis, primary tumor growth and lung metastasis in murine C26 colon adenocarcinoma [25]. These findings strongly support a role of MMP-1 in promoting tumor progression through the angiogenic processes. Our study showed that *CD26* was positively associated with *MMP1* expression in CRC cell line and clinical CRC samples, and our cell-line experiments demonstrated that CD26 functionally regulated the level of *MMP1*. We believe that MMP1 is involved in CD26-regulated angiogenesis and metastasis.

Our data further suggested that CD26 regulated *MMP1* through *CAV1*. CAV1 is an essential constituent protein of specialized membrane invaginations, referred to as caveolae. Loss of CAV1 was frequently observed in various types of malignancies including colon cancer [26], in which CAV-1 silencing results in activation of multiple survival signals including Src, EGFR, HER2, and the mitogen-activated protein kinase cascade and promotion of cancer transformation and initiation [27]. We showed that the *CAV1* level was lower in CD26+ HT29 cells, and its protein expression was negatively regulated by CD26 in CRC cell lines. More importantly, overexpression of CAV1 in shCD26 cells abrogated the MMP1 induction effect by CD26 overexpression, suggesting that CAV1 is essential for CD26-dependent regulation of *MMP1*.

This study demonstrated the functional roles of CD26 in inducing CRC migration, invasion, angiogenesis and metastasis and identified the potential involvement of *MMP1* and *CAV1* in such a process. CD26 is an attractive therapeutic target for combating tumor progression to improve the prognosis of CRC patients.

## 4. Materials and Methods

### 4.1. Cell Lines, Culture Conditions and Reagents

The human colorectal cancer cell lines HCT116 (MSI, Duke stage C, highly metastatic), HT29 (MSS, Duke stage C, metastatic), SW480 (MSS, Duke stage B, metastatic) and DLD1 (MSI, Duke stage C, weakly metastatic) were purchased from American Type Culture Collection (ATCC, Manassas, VA, USA). HCT116, HT29 and SW480 were routinely maintained in Dulbecco’s Modified Eagle’s Medium (Invitrogen, Carlsbad, CA, USA) supplemented with 10% FBS and 1% PS (Invitrogen). DLD1 was routinely maintained in Roswell Park Memorial Institute medium 1640 (Invitrogen) supplemented with 10% FBS and 1% PS (Invitrogen). Cells were incubated at 37 °C in a 5% CO_2_ air-humidified atmosphere. 

These cell lines were chosen due to the highest or lowest CD26+ proportion (Figure 1A). 

### 4.2. Fluorescence-Activated Cell Sorting (FACS)

One million CRC cells were washed once with 1× phosphate buffered saline (PBS).

Following centrifugation, cells were resuspended in 100 µL 1× staining buffer. Cells were stained by incubation with 5 µL CD26-PE/Cy5 antibody (1:20, Cat# 302708, Biolegend, Montgomery, TX, USA) for 15 min at room temperature according to the manufacturer’s recommendation. Samples were washed three times followed by resuspension in 500 µL 1× staining buffer (DMEM containing 2 mM EDTA) followed by flow cytometry analysis. Cells stained with non-specific antibodies (Cat# 400218, Biolegend) served as the isotypic control. Flow cytometry analysis was performed by the FACSCalibur flow cytometer and data were analyzed using the Flowjo software. For cell sorting experiment, after resuspension in staining buffer, cells were loaded into a cell sorter for isolation of cell subpopulations by the FACSAriaI cell sorter and data were analyzed using the Flowjo software. To increase stringency, only cells with the top and lowest 5% expression of cell surface molecule were gated for FACS.

### 4.3. Immunofluorescent Assay

HCT116 cells which showed the high CD26+ proportion were attached to the slides by the cytospin, then fixed with 4% formaldehyde for 15 min at room temperature. The fixed cells were washed with PBS three times, blocked in the blocking buffer (1× PBS/5% normal rabbit serum/0.3% Triton™ X-100 buffer) for 60 min, followed by overnight incubation with the primary antibodies against CD26 (Cat# ab28340, Abcam) or E-cadherin (Cat# 3915, Cell Signaling Technology, Danvers, MA, USA) at 4 °C. After washing with PBS, the specimen was incubated with fluorochrome-conjugated secondary antibody (1:1000, Cat# 4413, Cell Signaling Technology; Cat# A48254, ThermoFisher, Waltham, MA, USA) for 2 h at room temperature. Finally, the specimen was mounted with Prolong Gold Antifade Reagent (Invitrogen). Fluorescent signal was detected and captured by the confocal microscope (Carl Zeiss LSM 710, Oberkochen, Germany).

### 4.4. Transient and Stable Transfection

*CD26* expression was transiently down-regulated using predesigned siRNA duplex directly against *CD26* (Invitrogen) (sense: 5′-AAA GAU UCC UUC CUC CUG GCA-UUCC-3′ and anti-sense 5′-GGA AUG CCA GGA GGA AGG AAU CUU U-3′). A non-targeting siRNA (Invitrogen) was used as a negative control. Colorectal cancer cells were transfected with the annealed siRNA for 24 to 72 h using Lipofectamine 2000 (Invitrogen). pRS retroviral vector with customized sequence, the same as above, against *CD26* molecule was synthesized from Origene (Rockville, MD, USA). pRS retroviral vector with non-effective 29-mer scramble sequence was used as a control. Procedures for viral packaging, precipitation and stable transfection were previously mentioned.

### 4.5. Wound Healing Assay

CRC cells were cultured to 100% confluence in the 6-well culture plate. On the day of the assay, a wound across the well was created by a sterile yellow pipet tip. Cells were rinsed gently with PBS followed by replacement with completed media. Pictures were taken using phase-contrast light microscope at 0 h, 24 h and 48 h. All experiments were performed in triplicate.

### 4.6. Migration and Invasion Assay

For migration assays, 5 × 10^4^ to 1 × 10^5^ colorectal cancer cells were seeded onto the top chamber with non-coated membrane (BD Labware, San Diego, CA, USA). For invasion assays, 1 × 10^5^ colorectal cancer cells were plated in the top chamber with Matrigel-coated membrane (BD Biosciences, San Diego, CA, USA). For both assays, cells were resuspended in 250 μL of DMEM with 1% FBS and placed in the upper chamber, and DMEM with 10% FBS was used as a chemo-attractant in the lower chamber. Cells were incubated for 24 h at 37 °C in a 5% CO_2_ air-humidified atmosphere. Cells that did not migrate or invade through the pores of the upper chamber were removed by a cotton swab. Cells attached on the surface of the lower chamber were stained with 0.1% crystal violet before counted. For counting, each membrane was equally divided into 12 sections. Number of cells in 5 random sections were counted to determine the total number of cells migrated or invaded. Percentage of cells migrated or invaded was calculated as (No. of cells migrated/invaded ÷ total no. of cells seeded) × 100%. Each experiment was performed in duplicate, and results were presented as the mean ± SD of three independent experiments.

### 4.7. Animal Studies

The study protocol for performing in vivo assays in mice was described previously [28]. HT29 shCD26 or scramble control cells were subcutaneously injected into flank regions of nude mice and tumors were allowed to grow for 1 month, then the subcutaneous tumors were excised, cut into 1 mm^3^ pieces and orthotopically implanted onto the cecal wall of NOD/SICD mice. After 2 months the mice were euthanized. The primary tumors and livers were excised, fixed in formalin and then embedded in paraffin for further H&E and IHC staining to determine the presence of metastasis. The protocol is approved by and conducted in the accordance with the Committee of the Use of Live Animals in Teaching and Research at the University of Hong Kong.

### 4.8. Expression Array Analysis

Gene expression profiles of FACS-sorted CD26+ and CD26− subpopulation of HT29 cells were investigated using the Affymetrix human genome U133 Plus 2.0 GeneChip (Affymetrix, Waltham, MA, USA). Experiments were carried out at the Centre for Genomic Sciences of Faculty of Medicine, the University of Hong Kong. Data generated was analyzed using Genespring GX (Affymetrix) and Genomatix Genome Analyzer (Genomatix, Munich, Germany). Differentially expressed genes between HT29 CD26+ and CD26− cells were identified. Array data were deposited at the Gene Expression Omnibus (GEO accession no.: GSE192909).

### 4.9. RNA Extraction, cDNA Synthesis and Quantitative PCR

Total cellular RNA was extracted using PureLink^®^ RNA Mini Kit (Ambion, Waltham, MA, USA), and cDNA was synthesized with the PrimeScript Reverse Transcriptase (Takara, Shiga, Japan) according to the manufacturers’ instructions. Quantitative PCR (qPCR) was performed using the ABI 7900 Fast Sequence Detection Instruments (Applied Biosystems, Waltham, MA, USA). The expression of GAPDH was used to normalize that of the target genes. The 2^−ΔΔCt^ method was used to determine the relative fold differences among samples. Primers used for qPCR analysis were ordered from Integrated DNA Technologies (IDT, Singapore 117610, Republic of Singapore). Sequences of the primers are mentioned as below: *CD26*, Forward 5′-ATG GAC GGG GAA AGA AGA TA-3′, Reverse 5′-TTT ACA GTT GGA TTC ACA GCT C-3; *MMP1*, Forward 5′-ATG AAG CAG CCC AGA TGT GGA G-3′, Reverse 5′-TGG TCC ACA TCT GCT CTT GGC A-3; *CAV-1*, Forward 5′- CCA AGG AGA TCG ACC TGG TCA A-3′, Reverse 5′- GCC GTC AAA ACT GTG TGT CCC T-3; GAPDH, Forward 5′- GTC TCC TCT GAC TTC AAC AGC G-3′, Reverse 5′- ACC ACC CTG TTG CTG TAG CCA A-3.

### 4.10. Immunohistochemistry Staining

Mouse tissue samples were fixed in formalin, embedded in paraffin, cut into 5μm-thick sections by microtome, and mounted onto the slides coated with 3-aminopropryltriethoxysilance in acetone. Paraffin was removed by washing the sections three times in xylene and rehydration was achieved by washing the sections in serial dilutions of ethanol. Antigen retrieval was performed by microwave treatment for 10 min at 95 °C in citrate buffer. Biotin blocking kit was used for inhibiting the endogenous peroxidase and biotin activities. Afterwards, sections were blocked with horse serum for 30 min followed by incubating with the primary antibodies against CD26 (1:100, Cat#28340, Abcam, Cambridge, United Kingdom), Ki67 (1:100, Cat# M7240, Dako, Carpinteria, CA, USA) or VEGF (1:50, Cat# M7273, Dako) overnight at 4 °C in a moist chamber. The slides were then rinsed with TBS-T and TBS, followed by probing with biotinylated secondary antibody for an hour. Slides were then subsequently rinsed with TBS-T and TBS, and probed with avidin-horseradish peroxidase (Dako) for an hour. Signal was visualized by using DAB substrate-chromogen system. Lastly, nucleus was counterstained with hematoxylin and sections were mounted in DPX mountant. IHC scores were based upon the products of percentage positive cells multiplied by stain intensity (0 = negative, 1 = weak, 2 = moderate, 3 = strong) in three different high-power fields (400×) for each tumor section. To evaluate the immunostaining intensity, each slide was examined on a microscope. Representative 400× magnification fields of at least 100 tumor cells were selected and photographed.

Investigation of angiogenesis was based on H&E staining in accordance with protocol described in a previous study [29] and VEGF staining. The total number of blood vessels was determined in H&E-stained sections in high-power microscopic fields (×40) by counting vascular structures based on typical morphological appearance and the presence of the counted vascular lumen and intraluminal red blood cells. All visible blood vessels were counted in 8 random fields.

### 4.11. Statistical Analyses

Differences between groups were analyzed using the Student’s *t*-test or fisher exact test. All statistical graphs analyses were conducted were generated using GraphPad Prism 5.0, SPSS Statistics 20.0 or SigmaPlot 10.0. A *p*-value < 0.05 was regarded as statistically significant. 

## Data Availability

Array data were deposited at the Gene Expression Omnibus (GEO accession no.: GSE192909).

## Supplementary Materials

The following supporting information can be downloaded at: https://www.mdpi.com/article/10.3390/ijms23031181/s1.
